# The prevalence of job stressors among nurses in private in vitro fertilization (IVF) centres

**DOI:** 10.1002/nop2.187

**Published:** 2018-07-17

**Authors:** Le Dang Khoa, Tran Nhat Quang, Dang Quang Vinh, Nguyen Thi Ngoc Anh, Ho Manh Tuong, Kirsty Foster

**Affiliations:** ^1^ My Duc Hospital Ho Chi Minh Vietnam; ^2^ Faculty of Public Health University of Medicine and Pharmacy Ho Chi Minh Vietnam; ^3^ Research Center of Genetics & Reproductive Health School of Medicine—Vietnam National University Vietnam; ^4^ Office for Global Health, The University of Sydney Medical School of Medicine and Northern Clinical School New South Wales Australia

**Keywords:** IVF, job stress, medical workers, nurse, nursing, workforce

## Abstract

**Aim:**

The primary aim of this study was to identify the level of stress and the stressors having an impact on nurses compared with other medical workers in private IVF centres.

**Background:**

Stressful working conditions can an adversely affect not only the health and well‐being of health professionals but also subsequently to patient outcomes if care is given to infertile couples. This is of relevance particularly in view of Vietnam's recent economic growth and the increase in the number of private IVF centres. This is the first study looking at the levels of stress experienced by health workers (especially nurses) providing IVF services.

**Design:**

A cross‐sectional survey.

**Methods:**

All health workers in seven IVF Clinics in HCMC were invited to complete an Occupational Stress Index (OSI) questionnaire.

**Results:**

Of the invited 131 medical professionals, 105 (80%) completed the confidential self‐administered questionnaire. Thirty‐five participants (33.3%) were nurses, 19 (18.1%) were doctors and 51 (48.6%) were lab technicians. Approximately two‐thirds reported not having children (67.6%), half (50.48%) married and three‐quarters (76.2%) were women, with a significant difference by medical worker group (*p* < 0.05). Among the three groups, nurses have higher occupational stress index score compared with the others. The OSI score only had a strong relationship with the “high demand” (*p* < 0.001). Some demographic variables (e.g., income, long working hours, education level) statistically represented the high significant source of job stress.

## 1 INTRODUCTION

Many studies have shown that levels of distress at work are high in healthcare workers (Portoghese, Galletta, Coppola, Finco, & Campagna, 2014; Raiger, 2005; Ramirez, Graham, Richards, Cull, & Gregory, 1996). A variety of stress factors in the workplace, such as increased workload, emotional response to suffering/dying patients and organizational problems and conflicts, has been increasing the risk of distress for years among healthcare workers (McNeely, 2005). The negative effects of job stress on nurses have recently received increased attention. There is a wide range of potentially stressful situations in the workplace where the job stress could occur in nursing due to high workload, role conflict, limited staffing resources (Hsu, Chen, Yu, & Lou, 2010; Najimi, Goudarzi, & Sharifirad, 2012). Many findings have shown that the less job stress nurses have, the more job satisfaction, organizational commitment and the greater likelihood of turnover intentions they have (Garrosa, Moreno‐Jimenez, Liang, & Gonzalez, 2008; McGowan, 2001; Walker, 2008; Yeh, Ko, Chang, & Chen, 2007). In addition, working in very stressful environments with minimal control and social support from colleagues may also have a negative effect on patient safety (Berland, Natvig, & Gundersen, 2008).

Moreover, in vitro fertilization (IVF) centres are known to be particularly stressful (Costantini‐Ferrando, Joseph‐Sohan, Grill, Rauch, & Spandorfer, 2016; Harata et al., 2012). The high emotional content of consultations involved in the management of infertile couples is a contributory factor to the stress (Greenfeld, 1997; Oddens, den Tonkelaar, & Nieuwenhuyse, 1999). The unpredictable outcome of the treatment is another major stress‐inducing agent, more likely to evoke feelings of depression (Dunkel‐Schetter & Lobel, 1991). Stressful working conditions can adversely affect not only the health and well‐being of health professionals but also subsequently to patient outcomes (Halm et al., 2005; Leiter, Harvie, & Frizzell, 1998; Vahey, Aiken, Sloane, Clarke, & Vargas, 2004) if care is being given to infertile couples.

This is of relevance particularly in view of Vietnam's recent economic growth and the increase in the number of private IVF centres. To our knowledge, this is the first study looking at the levels of stress experienced by health workers (especially nurses) providing IVF services.

## 2 THE STUDY

### 2.1 Aims

The primary aim of this study was to identify the level of stress and the stressors having an impact on nurses compared with other medical workers (including physicians and IVF laboratory technicians) in private IVF centres to inform the development of strategies to benefit staff well‐being and quality of care for patients.

### 2.2 Design

A cross‐sectional study based on the survey design.

### 2.3 Participants

From August 2016 to November 2016, all employees involved in seven private IVF centres in South Vietnam were invited to participate in the study. Only permanent employees of these IVF centres were included. Interns, medical students, residence, collaborating staffs and university‐employed clinical lecturers were excluded.

### 2.4 Data collection

Information about the study was included with the invitation letter and those who were willing to participate completed an Occupational Stress Index (OSI) questionnaire. Completion of the questionnaire was deemed to signify consent to participate in the study. The medical workers themselves actually filled out the three specific OSI questionnaires, such as nurses‐specific OSI, physician‐specific OSI and generic OSI, tailored respectively to nurse, physician and IVF laboratory technician.

OSI questionnaires were completed by participants without the intervention of the researchers after the participants were fully informed about the aims and methods of the study. These questionnaires were usually conducted at lunchtime or after working hours. The OSI of every participant was given a unique number on the questionnaire. Principle Investigator (PI) kept a list of names and form numbers. The PI attended again the following week and collected completed OSI by recruiting late starters and any new medical workers. Then gentle reminders to complete were sent to participants by SMS. Research assistants chased up late forms on behalf of PI.

### 2.5 Ethical considerations

The study was reviewed by Scientific Board of Research Center for Genetics and Reproductive Health (CGRH)—National University and was conducted after the Ethics Committee approval of My Duc Hospital had been granted.

### 2.6 Data analysis

The Occupation Stressor Index (Belkic, 2000; Belkic & Nedic, 2007, 2012 ; Levi et al., 2000; Nedic, Belkic, Filipovic, & Jocic, 2010), a two‐dimensional matrix, was defined by the vertical axis composed of levels of information transmission (including input, central decision‐making, output, general) and the stressor aspects placed along the horizontal axis. Each element in the OSI was scored from 0 to 2 (0: “not present,” 2: “strongly present”) and summed to give total scores under each domain. The domains are underload, high demand, strictness, external time pressure, aversive physical exposures, symbolic aversiveness (or avoidance) and conflict (or uncertainty). Summed scores for the aspects and the total OSI were calculated according to this framework (Belkić & Savić, 2013).

The data was stored in Microsoft Excel and the stress index scores were calculated for each participant using the scoresheet for each of the three specific OSI questionnaires. Categorical variables were reported as frequencies (percentages) and continuous variables were summarized as means (standard deviations). Means among the three medical workers were analysed by one‐way analysis of variance (ANOVA). Between‐group differences in discrete variables were assessed by using the Chi‐square test. If expected cell size was under five, the Fisher's exact test was used with groups take two at a time. Tukey's HSD post hoc test was calculated for each mean comparison. A correlation matrix was used to investigate the dependence between multiple variables at the same time. All analyses were performed using the R statistical packages. A *p*‐values <0.05 was considered statistically significant.

### 2.7 Validity, reliability and rigour

The OSI is an international validated survey tool developed and tailored specifically for professionals (Belkic, 2000; Belkic & Nedic, 2007, 2012 ; Levi et al., 2000; Nedic et al., 2010) to assess precisely levels of known stressors experienced by medical workers (including nurses, doctors, IVF laboratory technicians). The English versions of OSI questionnaire were translated into Vietnamese by using double translation technique approved by original developer in the US for ensuring linguistic validity.

Cronbach's alpha was also calculated for variable elements of each aspect of the OSI and was used to measure the strength of internal consistency of OSI aspects. The Cronbach alpha of OSI aspects which ranged from 0.75 to 0.79 in our findings showed acceptable reliability.

## 3 RESULTS

**Figure 1:**
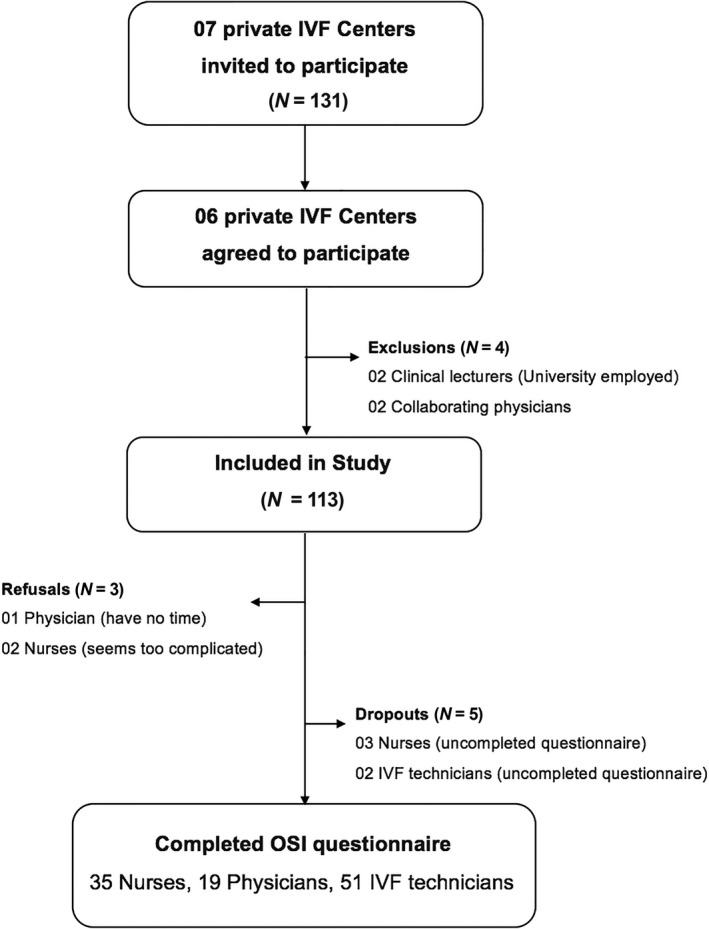
Recruitment process flowchart

Of the invited 131 medical professionals, 105 (80%) completed the confidential self‐administered questionnaire (Figure 1). Thirty‐five participants (33.3%) were nurses, 19 (18.1%) were doctors and 51 (48.6%) were IVF laboratory technicians. Approximately two‐thirds reported not having children (67.6%, 71), half (50.48%, 53) were married and three‐quarters (76.2%, 80) were women, with a significant difference by medical worker group (*p* < 0.05). Among the three groups, nurses significantly had lower education levels and lower income compared with the others. Further demographic characteristics of all participants are given in Table 1. Some of them (e.g., income, long working hours, education level, autonomous workspace) statistically represented the high significant source of job stress.

**Table 1:** Baseline characteristics of the three medical worker groups

	All (*N* = 105)	Nurse (*N* = 35)	Doctor (*N* = 19)	IVF technician (*N* = 51)	*p*‐values
	Mean (*SD*) or *N* (%)	Mean (*SD*) or *N* (%)	Mean (*SD*) or *N* (%)	Mean (*SD*) or *N* (%)	
Age	31.06 (8.15)	29.83 (8.39)	38.26 (6.03)	29.22 (7.28)	<0.001
Distance (home‐to‐hospital)	10.00 (7.95)	10.51 (8.09)	9.53 (4.82)	9.82 (8.84)	0.889
Number of work years	6.55 (6.16)	7.40 (7.61)	11.95 (4.64)	3.96 (3.64)	<0.001
Homeowner	33 (31.43)	9 (25.71)	14 (73.68)	10 (19.61)	<0.001
Transportation					0.878*
Motorcycle	90 (85.71)	30 (85.71)	17 (89.47)	43 (84.31)	
Other	15 (14.29)	05 (14.29)	02 (10.53)	08 (15.69)	
Education					<0.001
Postgraduate	29 (27.62)	0 (0.00)	14 (73.68)	15 (29.41)	
Undergraduate	76 (72.38)	35 (100)	05 (26.32)	36 (70.59)	
Income (USD/year)					<0.001*
Under 10,000	70 (66.67)	33 (94.29)	05 (26.32)	32 (62.75)	
10,000–19,000	20 (19.05)	02 (5.714)	05 (26.32)	13 (25.49)	
20,000–30,000	9 (8.57)	0 (0.00)	04 (21.05)	05 (9.80)	
30,000–40,000	2 (1.90)	0 (0.00)	01 (5.26)	01 (1.96)	
Over 40,000	4 (3.81)	0 (0.00)	04 (21.05)	0 (0.00)	
Long work hours/week					0.023*
Under 48	13 (12.38)	05 (14.29)	06 (31.58)	02 (3.92)	
48–60	69 (65.71)	20 (57.14)	11 (57.89)	38 (74.51)	
Over 60	23 (21.90)	10 (28.57)	02 (10.53)	11 (21.57)	
OSI range (total score)					0.023*
Under 70	52 (49.52)	11 (31.43)	09 (47.37)	32 (62.75)	
70–80	41 (39.05)	16 (45.71)	09 (47.37)	16 (31.37)	
80–90	12 (11.43)	08 (22.86)	01 (05.26)	03 (05.88)	
Over 90	0 (00.00)	00 (00.00)	00 (00.00)	00 (00.00)	

*Note. *

*Fisher's exact test.

**Figure 2:**
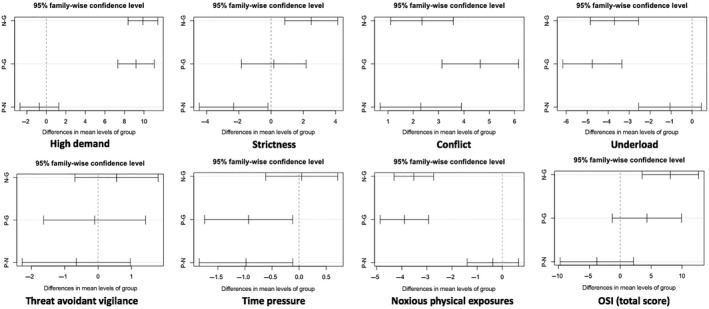
Tukey’s HSD post hoc analysis for differences among the three groups (P: Physician, N: Nurse, G: IVF lab technician)

The OSI scores were significantly different among three groups with the exception of the threat avoidant vigilance, with nurses scoring most highly (see Table 2). Figure 2 shows where the differences in mean levels of OSI aspects occurred among the three groups. In nurses, the OSI score only had a moderate one with the noxious physical exposures, the threat avoidant vigilance and the conflict (*p* < 0.05) and had a strong uphill linear pattern with the high demand (*p* < 0.001). In IVF laboratory technicians, the threat avoidant vigilance and the conflict had a strong relationship with OSI score (*p* < 0.001) while the strictness and the noxious physical exposures had a moderate one (*p* < 0.001). Otherwise, the OSI score had a strong relationship with the high demand (*p* < 0.05) and had a moderate on with the conflict (*p* = 0.05) and the underload (*p* = 0.16) in the physician group. The detailed stressors between medical worker groups were clearly analysed in Table 3.

**Table 2:** Occupational stress index aspects of the three medical worker groups

Aspect of OSI	Cronbach alpha	All (*N* = 105)	Nurse (*N* = 35)	Doctor (*N* = 19)	IVF technician (*N* = 51)	*p*‐values
		Mean (*SD*)	Mean (*SD*)	Mean (*SD*)	Mean (*SD*)	
High demand	0.75	24.24 (5.65)	29.17 (2.80)	28.46 (2.71)	19.27 (3.08)	<0.001
Strictness	0.79	12.15 (3.32)	13.79 (2.79)	11.46 (2.12)	11.29 (3.64)	0.001
Conflict/uncertainty	0.77	9.85 (2.94)	10.57 (2.03)	12.87 (2.07)	8.23 (2.66)	<0.001
Underload	0.77	4.82 (3.01)	3.21 (1.73)	2.16 (1.41)	6.91 (2.67)	<0.001
Threat avoidant vigilance	0.78	8.24 (2.38)	8.63 (2.22)	7.97 (1.88)	8.08 (2.65)	0.5
Extrinsic time pressure	0.79	6.62 (1.31)	6.82 (1.11)	5.84 (0.83)	6.78 (1.49)	0.015
Noxious physical exposures	0.78	3.04 (2.37)	1.40 (1.24)	1.03 (0.98)	4.92 (1.81)	<0.001
OSI (Total score)	0.79	68.96 (9.42)	73.59 (6.85)	69.79 (6.24)	65.48 (10.53)	<0.001

**Table 3:** A detailed analysis for stressors among three groups

	All (*N* = 105)	Nurse (*N* = 35)	Doctor (*N* = 19)	IVF technicians (*N* = 51)	*p*‐value
	*N* (%)	*N* (%)	*N* (%)	*N* (%)	
**Underload**					
Fixed pay	50 (47.62)	16 (45.71)	3 (15.79)	31 (60.78)	<0.001*
Inadequate pay					<0.001*
Totally inadequate	9 (8.57)	5 (14.29)	0 (0.00)	4 (7.84)	
Just barely covers expense	48 (45.71)	19 (54.29)	1 (5.26)	28 (54.90)	
Promotion					0.444*
Yes	80 (76.19)	24 (68.57)	15 (78.95)	41 (80.39)	
No	25 (23.81)	11 (31.43)	4 (21.05)	10 (19.61)	
Recognition of good work					0.852*
Definitely yes	41 (39.05)	14 (40.00)	8 (42.11)	19 (37.25)	
Yes, to some extent	43 (40.95)	15 (42.86)	8 (42.11)	20 (39.22)	
Not very much	20 (19.05)	5 (14.29)	3 (15.79)	12 (23.53)	
Not at all	1 (0.95)	1 (2.86)	0 (0.00)	0 (0.00)	
**High demand**					
Piece rate					<0.001*
Fixed pay	50 (47.61)	16 (45.71)	3 (15.79)	31 (60.78)	
Group work	23 (21.91)	12 (34.29)	3 (15.79)	8 (15.69)	
By individual work	32 (30.48)	7 (20.00)	13 (68.42)	12 (23.53)	
Long work hours					0.022*
Not more than 48 hr/week	13 (12.38)	5 (14.29)	6 (31.58)	2 (3.92)	
48 to 60 hr/week	69 (65.71)	20 (57.14)	11 (57.89)	38 (74.51)	
More than 60 hr/week	23 (21.90)	10 (28.57)	2 (10.53)	11 (21.57)	
Two or more jobs					0.486*
Yes	12 (11.43)	3 (8.57)	3 (15.79)	6 (11.76)	
No	93 (88.57)	32 (91.43)	16 (84.21)	45 (88.24)	
Rest breaks					0.022*
Never	4 (3.81)	3 (8.57)	0 (0.00)	1 (1.96)	
Rarely	9 (8.57)	6 (17.14)	0 (0.00)	3 (5.88)	
Occasionally	75 (71.43)	24 (68.57)	13 (68.42)	38 (74.51)	
Frequently	17 (16.19)	2 (5.71)	6 (31.58)	9 (17.65)	
Night shift/irregular work					<0.001*
Yes	11 (10.48)	1 (2.86)	10 (52.63)	0 (0.00)	
No	94 (89.52)	34 (97.14)	9 (47.37)	51 (100)	
Insufficient paid vacation					0.058
3–4 weeks	1 (0.95)	0 (0.00)	1 (5.26)	0 (0.00)	
2 weeks	98 (93.33)	35 (100)	17 (89.47)	46 (90.20)	
Less than 2 weeks	6 (5.72)	0 (0.00)	1 (5.26)	5 (9.80)	
**Strictness**					
Fixed posture					<0.001*
Mobile	59 (56.19)	17 (48.57)	10 (52.63)	32 (62.75)	
Main single posture, but free to move	22 (20.95)	4 (11.43)	0 (0.00)	18 (35.29)	
Fixed body position, constrained motion	24 (22.86)	14 (40.00)	9 (47.37)	1 (1.96)	
Window—less area					<0.001*
With a direct window	31 (29.52)	13 (37.14)	5 (26.32)	13 (25.49)	
With an indirect window	22 (20.950	11 (31.43)	5 (26.32)	6 (11.76)	
Without a window	52 (49.52)	11 (31.43)	9 (47.37)	32 (62.75)	
Autonomous workspace					<0.001*
Yes	10 (9.52)	4 (11.43)	6 (31.58)	0 (0.00)	
No	95 (90.48)	31 (88.57)	13 (68.42)	51 (100)	
Chance to take time off from work					0.036*
No problem (to take time off)	13 (12.38)	0 (0.00)	3 (15.79)	10 (19.61)	
A little difficult	27 (25.71)	8 (22.86)	7 (36.84)	12 (23.53)	
Somewhat difficult	54 (51.43)	23 (65.71)	7 (36.84)	24 (47.06)	
Very difficult	11 (10.48)	4 (11.43)	2 (10.53)	5 (9.80)	
Low influence over schedule					0.012*
Complete influence	1 (0.95)	0 (0.00)	1 (5.26)	0 (0.00)	
Major influence	37 (35.24)	8 (22.86)	11 (57.90)	18 (35.29)	
A little influence	54 (51.43)	23 (65.71)	7 (36.84)	24 (47.06)	
No influence	13 (12.38)	4 (11.43)	0 (0.00)	9 (17.65)	
Low influence over with whom works					0.156*
Complete influence	16 (15.24)	8 (22.86)	3 (15.79)	5 (9.80)	
Major influence	36 (34.29)	11 (31.43)	5 (26.32)	20 (39.22)	
A little influence	20 (19.05)	7 (20.00)	7 (36.84)	6 (11.77)	
No influence	33 (31.43)	9 (25.71)	4 (21.05)	20 (39.22)	
Low influence over tasks					0.043*
Complete influence	30 (28.57)	10 (28.57)	3 (15.79)	17 (33.33)	
Major influence	45 (42.86)	14 (40.00)	9 (47.37)	22 (43.14)	
A little influence	18 (17.14)	9 (25.71)	6 (31.58)	3 (5.88)	
No influence	12 (11.43)	2 (5.71)	1 (5.26)	9 (17.65)	
Low influence over policy					0.691*
Complete influence	22 (20.95)	9 (25.71)	4 (21.05)	9 (17.65)	
Major influence	37 (35.24)	12 (34.29)	8 (42.11)	17 (33.33)	
A little influence	27 (25.71)	10 (28.57)	5 (26.32)	12 (23.53)	
No influence	19 (18.10)	4 (11.43)	2 (10.53)	13 (25.49)	
**Extrinsic time pressure**					
Deadline pressure					0.104*
Never	6 (5.71)	4 (11.43)	1 (5.26)	1 (1.96)	
Rarely	11 (10.48)	6 (17.14)	3 (15.79)	2 (3.92)	
Occasionally	41 (39.05)	13 (37.14)	8 (42.11)	20 (39.22)	
Frequently	47 (44.76)	12 (34.29)	7 (36.84)	28 (54.90)	
Speedup					0.412*
Rarely or never	9 (8.57)	3 (8.57)	1 (5.26)	5 (9.80)	
Certain periods of the month or year	73 (69.52)	23 (65.71)	15 (78.95)	35 (68.64)	
At least weekly but not daily	10 (9.52)	2 (5.71)	3 (15.79)	5 (9.80)	
Daily	13 (12.38)	7 (20.00)	0 (0.00)	6 (11.76)	
**Noxious exposure**					
Heat exposure					<0.001*
Not over 25°C	32 (30.48)	17 (48.57)	13 (68.42)	2 (3.92)	
Not over 30°C	58 (55.24)	13 (37.14)	4 (21.05)	41 (80.39)	
Over 30°C	15 (14.29)	5 (14.29)	2 (10.53)	8 (15.69)	
Cold exposure					0.061*
At least 20°C	91 (86.67)	27 (77.14)	19 (100)	45 (88.24)	
At least 18°C	13 (12.38)	8 (22.86)	0 (0.00)	5 (9.80)	
Under 18°C	1 (0.95)	0 (0.00)	0 (0.00)	1 (1.96)	
Noxious gases, fumes, dusts					<0.001
Never	48 (45.71)	25 (71.43)	15 (78.95)	8 (15.69)	
At least occasionally	57 (54.29)	10 (28.57)	4 (21.05)	43 (84.31)	
**Threat avoidant vigilance**					
Experienced accident at work					0.013
Yes	48 (45.71)	14 (40.00)	4 (21.05)	30 (58.82)	
No	57 (54.29)	21 (60.00)	15 (78.95)	21 (41.18)	
Witnessed accident at work					0.185*
Never heard about or witnessed	0 (0.00)	0 (0.00)	0 (0.00)	0 (0.00)	
Heard about but never witnessed	8 (7.62)	4 (11.43)	2 (10.53)	2 (3.92)	
Witnessed a serious accident at work	6 (5.71)	0 (0.00)	2 (10.53)	4 (7.84)	
Witnessed fatal accident at work	91 (86.67)	31 (88.57)	15 (78.95)	45 (88.24)	
Suicide among coworkers					0.495*
Yes	23 (21.90)	6 (17.14)	6 (31.58)	11 (21.57)	
No	82 (78.10)	29 (82.86)	13 (68.42)	40 (78.43)	
Litigation/testifying in court					0.140*
No feedback	2 (1.91)	2 (5.71)	0 (0.00)	0 (0.00)	
No	103 (98.10)	33 (94.29)	19 (100)	51 (100)	
Functioning emergency system					0.154*
Yes, and knows that it functions properly	24 (22.86)	7 (20.00)	2 (10.53)	15 (29.41)	
Yes, but does not know whether it functions properly	51 (48.57)	15 (42.86)	10 (52.63)	26 (50.98)	
No	30 (28.57)	13 (37.14)	7 (36.84)	10 (19.61)	
**Conflict/uncertainty**					
Emotionally charged work atmosphere					0.464*
No	21 (20.00)	7 (20.00)	4 (21.05)	10 (19.61)	
Minimal	63 (60.00)	24 (68.57)	12 (63.16)	27 (52.94)	
Occasionally	21 (20.00)	4 (11.43)	3 (15.79)	14 (27.45)	
Great deal of tension	0 (0.00)	0 (0.00)	0 (0.00)	0 (0.00)	
Lack of help with difficulties					<0.001*
Can count on help	73 (69.52)	33 (94.28)	15 (78.95)	25 (49.02)	
More often than not, can get help	28 (26.67)	1 (2.86)	3 (15.79)	24 (47.06)	
Can't really count on help	2 (1.90)	0 (0.00)	0 (0.00)	2 (3.92)	
Rarely of never can get help	2 (1.90)	1 (2.86)	1 (5.26)	0 (0.00)	
Opposition to career advancement					0.444*
Yes	80 (76.19)	24 (68.57)	15 (78.95)	41 (80.39)	
No	25 (23.81)	11 (31.43)	4 (21.05)	10 (19.61)	
Violations of behaviour norms/abuses of power					0.002*
Never	78 (74.29)	33 (94.28)	13 (68.42)	32 (62.75)	
Rarely	20 (19.05)	1 (2.86)	3 (15.79)	16 (31.37)	
Occasionally	7 (6.67)	1 (2.86)	3 (15.79)	3 (5.88)	
Frequently	0 (0.00)	0 (0.00)	0 (0.00)	0 (0.00)	
Lack of redress of grievance					0.02*
Redress can be done and is efficient and confidential	55 (52.38)	19 (54.29)	12 (63.16)	24 (47.06)	
In principle, yes but not effective or not confidential	29 (27.62)	5 (14.29)	5 (26.32)	19 (37.25)	
No opportunity to redress grievances	21 (20.00)	11 (31.42)	2 (10.53)	8 (15.69)	
Threat of job loss					0.475*
Yes	6 (5.71)	2 (5.71)	2 (10.53)	2 (3.92)	
No	99 (94.29)	33 (94.29)	17 (89.47)	49 (96.08)	
Job lacks coherence					<0.001*
Work tasks fit together and clear relation to the goals	55 (52.38)	7 (20.00)	2 (10.53)	46 (90.20)	
Some work tasks fit together, vague relation to the goals	48 (45.71)	28 (80.00)	17 (89.47)	3 (5.88)	
Disconnected work tasks, unclear relation to the goals	2 (1.90)	0 (0.00)	0 (0.00)	2 (3.92)	
**Hinder from giving adequate patient care**					
Lack of needed supplies (including medications)	4 (3.81)	2 (5.71)	2 (10.53)	0 (0.00)	
Lack of hospital beds	18 (17.14)	14 (40.00)	4 (21.05)	0 (0.00)	
Understaffing	15 (14.29)	10 (28.57)	4 (21.05)	1 (1.96)	
Administrative constraints to ordering needed supplies	6 (5.714)	5 (14.29)	1 (5.26)	0 (0.00)	
Language barriers with patients (lack of translators)	16 (15.24)	12 (34.29)	3 (15.79)	1 (1.96)	
Infrastructural problems	14 (13.33)	7 (20.00)	2 (10.53)	5 (9.80)	
Need for frequent patient transport under tenuous conditions	0 (0.00)	0 (0.00)	0 (0.00)	0 (0.00)	
Delay or inability to obtain medical records	7 (6.67)	3 (8.57)	4 (21.05)	0 (0.00)	
Difficulty in obtaining laboratory results	2 (1.91)	1 (2.86)	1 (5.26)	0 (0.00)	
Limitations in ordering tests	1 (0.95)	0 (0.00)	1 (5.26)	0 (0.00)	
Limitations on sending patients for consult	1 (0.95)	0 (0.00)	1 (5.26)	0 (0.00)	
Physical harm or injury at work	48 (45.71)	14 (40.00)	4 (21.05)	30 (58.82)	0.013
Colleague or staff at the any of the places where you have worked ever committed suicide	23 (21.9)	6 (17.14)	6 (31.58)	11 (21.57)	0.495*

*Note. *

*Fisher's exact test.

## 4 DISCUSSION

This is the first study to examine stressors for health workers in IVF Clinics. What we have shown is that there are clear differences between nurses, doctors and IVF technicians in terms of the factors which cause them stress and each group varies in the factors that ameliorate their stress. In the nurse group, it was found that (i) the mean OSI score was significantly higher when compared with each of the other groups (*p* < 0.001); (ii) the OSI score only had a strong relationship with the “high demand” (*p* < 0.001); (iii) Some demographic variables (e.g., income, long working hours, education level, autonomous workspace) statistically represented the high significant source of job stress.

This study confirms previous findings that night shift and long working hours are one aspect of “high demand,” which has a strong influence of on job stress (Cheng, Liou, Tsai, & Chang, 2015; Coffey, Skipper, & Jung, 1988; Kirkcaldy, Trimpop, & Cooper, 1997; Nabirye, Brown, Pryor, & Maples, 2011; van Wijk, 1997). Our results are also consistent with the findings of Hsiu–Yueh when the occupational stress differed significantly by salary level (Hsu et al., 2010). However, the findings of Garrett and McDaniel showed that demographic variables (e.g., education level, years of service) had no statistically significant relationship with job stress (Garrett & McDaniel, 2001).

As mentioned above, some demographic variables statistically represented the high significant source of job stress. We did a further linear regression analysis to check whether the significant difference between groups is caused by these demographics. The findings show that demographic variables were not the factors causing these significant different OSI scores between groups with *p*‐value >0.05. However, *R* ^2^, a statistical measure of how close the data are to the fitted regression line, is only around 0.148 (14.8% of the variability of the response data around its mean). Thus, the potential mechanism for the highest OSI score of nurses among three groups remains to be elucidated. There are few reasonable hypotheses. The lower incomes and longer working hours, compared with the other groups, maybe the main reasons. Being hindered from giving adequate patient care, more difficult to take time off from work and lower influence over schedule and lack of autonomous workspace may also contribute to higher OSI score (Table 1).

Belkic and colleagues found that the urgent intervention was needed when the prototypical total OSI scores for clinical cases involving mental health disorders was >90 (Belkić & Savić, 2013). However, all nurses in our study had OSI scores under 90. Firstly, it is possible that this is a real characteristic of this population, but this may also be a chance result. Secondly, the descriptive analysis of levels of occupational stress by demographic characteristics (age, education level, number of work years), conducted by Nabirye et al., indicated that the higher the nurses had progressed in the system, the more stressed they had progressed in the system, the more stressed they have become (Jordan, Khubchandani, & Wiblishauser, 2016; Nabirye et al., 2011). By contrast, our findings show that the nurses had the lowest level of education, but they have obtained the highest OSI scores. It is possible that the working environment which had high rates of promotion, recognition at work, rest breaks and support with difficulties may have lead to the under‐90‐OSI scores.

The most significant implication of this study concerns the improvement of nurses’ work environment in IVF field. Organizations wishing to give patients with a high quality and compassionate service are advised to consider working conditions which can cause stress to employees. There are many suggesting strategies to retain nurses or recruiting new nurses (Berliner & Ginzberg, 2002; Smith‐Stoner & Markley, 2007), but identifying ways to improve the work environment is still lacking. These may be in the way the workforce is managed or in the physical design and configuration of the workplace. Although the results can be generalized only to nurses in IVF settings, the findings may be helpful for other medical staff (physicians, IVF lab technicians) who are working together with nurses. Another significant contribution from this study is it is highlighting the need for future researchers to focus on the variables that had strong or moderate relationships with stress, for example, an aspect such as the threat avoidant vigilance. Research should be conducted to test the hypothesis that helps explain the relationship between accidents at work and job stress.

### 4.1 Limitations

Although the OSI is an international detailed and validated survey tool developed specifically for professionals, this study also has several limitations. First, the recall and nonresponse bias maybe occurred because the questionnaire measured only self‐reported data and was quite long with nine pages. Second, the study was only based on quantitative approach. Future studies should include a qualitative aspect to gain a deep understanding of these issues. Third, this study failed to differentiate normal nurses from the stress ones. The Vietnamese validated version of OSI questionnaire is needed to find the cut‐off score for appropriate and just‐in‐time intervention.

## 5 CONCLUSIONS

In the nurse group, the OSI score was significantly highest among the three groups. This points to the importance of “high demand” aspect which has a strong relationship with OSI score. The authors recommend that further studies involve not only nurses but also medical workers in IVF field to increase the generalizability of the findings.

## CONFLICT OF INTERESTS

No conflict of interest has been declared by the authors.

## AUTHOR CONTRIBUTIONS

Le Dang Khoa was involved in study design, execution, analysis, manuscript drafting and critical discussion. Kirsty Foster was involved in study design, execution, provided critical revision for important intellectual content. Tran Nhat Quang was involved in clinical data analysis, critical discussion. Ho Manh Tuong, Dang Quang Vinh and Nguyen Thi Ngoc Anh were involved in the execution, critical discussion. All authors approved the final version to be published.

All authors have agreed on the final version and meet at least one of the following criteria (recommended by the International Committee of Medical Journal Editors [https://www.icmje.org/recommendations/]):

- substantial contributions to conception and design, acquisition of data, or analysis and interpretation of data;
- drafting the article or revising it critically for important intellectual content.
